# Titanium Nitride as a Plasmonic Material from Near-Ultraviolet to Very-Long-Wavelength Infrared Range

**DOI:** 10.3390/ma14227095

**Published:** 2021-11-22

**Authors:** Jarosław Judek, Piotr Wróbel, Paweł Piotr Michałowski, Monika Ożga, Bartłomiej Witkowski, Aleksandra Seweryn, Michał Struzik, Cezariusz Jastrzębski, Krzysztof Zberecki

**Affiliations:** 1 Institute of Microelectronics and Optoelectronics, Warsaw University of Technology, Koszykowa 75, 00-662 Warsaw, Poland; 2 Faculty of Physics, University of Warsaw, Pasteura 5, 02-093 Warsaw, Poland; piotr.wrobel@fuw.edu.pl; 3 Łukasiewicz Research Network—Institute of Microelectronics and Photonics, Aleja Lotnikow 32/46, 02-668 Warsaw, Poland; pawel.michalowski@imif.lukasiewicz.gov.pl; 4 Institute of Physics, Polish Academy of Sciences, Aleja Lotników 32/46, 02-668 Warsaw, Poland; ozga@ifpan.edu.pl (M.O.); bwitkow@ifpan.edu.pl (B.W.); aseweryn@ifpan.edu.pl (A.S.); 5 Faculty of Physics, Warsaw University of Technology, Koszykowa 75, 00-662 Warsaw, Poland; michal.struzik@pw.edu.pl (M.S.); cezariusz.jastrzebski@pw.edu.pl (C.J.); krzysztof.zberecki@pw.edu.pl (K.Z.)

**Keywords:** photonics, plasmonics, titanium nitride, infrared range

## Abstract

Titanium nitride is a well-known conductive ceramic material that has recently experienced resumed attention because of its plasmonic properties comparable to metallic gold and silver. Thus, TiN is an attractive alternative for modern and future photonic applications that require compatibility with the Complementary Metal-Oxide-Semiconductor (CMOS) technology or improved resistance to temperatures or radiation. This work demonstrates that polycrystalline TiN*_x_* films sputtered on silicon at room temperature can exhibit plasmonic properties continuously from 400 nm up to 30 μm. The films’ composition, expressed as nitrogen to titanium ratio x and determined in the Secondary Ion Mass Spectroscopy (SIMS) experiment to be in the range of 0.84 to 1.21, is essential for optimizing the plasmonic properties. In the visible range, the dielectric function renders the interband optical transitions. For wavelengths longer than 800 nm, the optical properties of TiN*_x_* are well described by the Drude model modified by an additional Lorentz term, which has to be included for part of the samples. The ab initio calculations support the experimental results both in the visible and infra-red ranges; particularly, the existence of a very low energy optical transition is predicted. Some other minor features in the dielectric function observed for the longest wavelengths are suspected to be of phonon origin.

## 1. Introduction

Titanium nitride is a well-known high-temperature-stable harsh-environment-resistant conductive ceramic material readily used by the industry, mainly as a protective [1] or decorative coating [2]. Another field of application is in microelectronics, where titanium nitride acts as an efficient anti-diffusion layer [3], adhesion layer [4], and top-gate in field-effect transistors [5]. However, the most recent studies on TiN and other refractory metal nitrides like HfN or ZrN are related to their plasmonic nature, which is the ability to strongly interact with the light through coupling with surface plasma oscillations [6,7]. In this area, titanium nitride is considered an attractive alternative to gold and silver, nowadays standard materials used in plasmonic applications [8]. It is much more cost-effective, mechanically and thermally robust, and, importantly, compatible with the CMOS technology despite worse optical properties. Successful implementation of the titanium nitride as a plasmonic component in photonic devices has been demonstrated on the example of hyperbolic metamaterials working in the visible and infrared ranges [9,10], high-temperature-stable and irradiation-resistant broadband absorber [11], nanoantennas, or other nanostructures able to increase optical response [12,13,14,15,16,17], SERS (Surface-Enhanced Raman Spectroscopy spectroscopy) substrate [18], and remote optical temperature sensor [19]. Apart from applications, the current literature addresses issues related to optimizing the plasmonic properties [20,21,22,23,24,25,26] and the stability of the thin films [26,27,28]. Some quite modern reviews are also available [29,30].

This work shows and analyzes the complex dielectric function—a quantity entirely determining all optical properties of a solid, of stoichiometric and nonstoichiometric titanium nitride samples in the spectral range of 193 nm to 30 μm, with particular focus on the 800 nm–20 μm range. Literature scarcely reports optical properties of TiN for wavelengths longer than a few micrometers [31,32,33], and limited data are available in this range and only from reflectance [34] and FTIR (Fourier transform infrared) [16,35] measurements. Since the development of any functional plasmonic device requires knowledge of the optical properties of constituent materials, in the case of a lack of necessary data, adequate investigations have to be undertaken. The main scientific objective should resolve whether a Drude model can describe optical properties at longer wavelengths or some other physical phenomena such as low energy optical absorption, e.g., related to defects or phonons, have to be considered as well. Findings provided by this work, particularly reliable analytical models of stoichiometric and nonstoichiometric titanium nitride thin films valid in the range of 800 nm to 20 μm, are thus of fundamental importance for the development of CMOS-compatible plasmonic materials working in the infrared 3 μm to 5 μm and 8 μm to 12 μm wavebands, which are readily used, e.g., in the thermovision [36] and by gas sensors [37,38].

## 2. Materials, Methods and Models

### 2.1. Magnetron Sputtering

**Figure 1:**
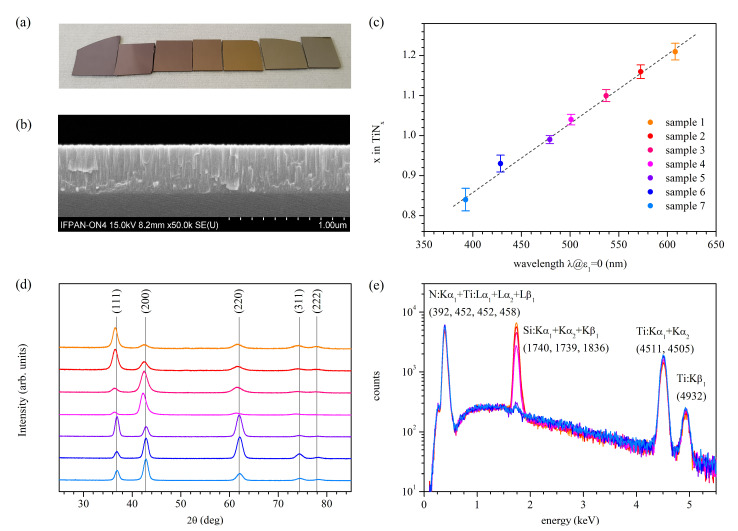
(**a**) Picture of one of the three series of samples; (**b**) SEM image of a cross-section of the stoichiometric sample; (**c**) composition of TiN*_x_* samples determined from SIMS experiment as a function of the wavelength, at which the real part of the dielectric function crosses zero; (**d**) GIXRD patterns proving that the crystal structure $\mathrm{Fm}\bar{3}m$ is preserved despite changes in composition; (**e**) EDS spectra proving that there are no other elements than Ti and N; the Si line comes from the substrate.

Titanium nitride thin films were deposited on the (100) silicon substrate using a pulsed-DC reactive magnetron sputtering system at room temperature (Plasmalab System 400, Oxford Instruments, Bristol, UK). We used pure titanium as the target material and argon as a primary working gas. The addition of nitrogen made the process reactive. The stoichiometry of TiN*_x_* samples was controlled by the ratio of nitrogen flux to argon flux while keeping the total flux approximately constant. Provided power, deposition time, pressure, and other details of the deposition process, except gas fluxes, were kept constant for all the samples. For our experiments, we fabricated three of the same series of seven samples each. A picture of one of the series is shown in Figure 1a. The first sample is the most nitrogen-rich, the fifth is almost stoichiometric, and the last is the most nitrogen-deficient.

### 2.2. SIMS

The exact composition of TiN*_x_* samples was determined from the SIMS experiment using IMS SC Ultra instrument (CAMECA, Gennevilliers Cedex, France) under ultra-high vacuum, typically 4 × 10^−10^ mbar. Cs+ primary beam was rastered over 250 × 250 µm^2^, and the analysis area was limited to 200 × 200 µm^2^. The intensity of the primary beam was 40 nA, and the impact energy was 7 keV. Positive ions detection mode was used in the experiments, and thus all species were measured as CsX+ cluster ions. To determine the composition of TiN*_x_* CsN+/CsTi+, the signals ratio was calibrated with a reference sample (stoichiometric TiN). Twenty independent measurements were performed for each sample. The result of the measurement is the mean value of all measurements, whereas the uncertainty is the standard deviation value.

### 2.3. XRD

X-ray diffraction data (XRD) were collected at room temperature on a Empyrean X-ray diffractometer (Malvern Panalytical, Malvern, UK), with a PIXcel3D detector (Malvern Panalytical, Malvern, UK), using Ni filtered Cu-Ka radiation at an accelerating voltage of 45 kV with grazing incidence geometry and an incidence angle of *α*_i_ = 0.4°. Data were collected at room temperature in the 2*θ* angle range of 25° to 85°, in steps of 0.0263° with an effective scan time of 300 s per step. The incidence angle has been determined experimentally in a series of reference measurements with a varying value of the incidence angle (Supplementary Material, Figure S1).

### 2.4. Raman Spectroscopy

Raman measurements of the TiN*_x_* samples were performed under normal conditions using an InVia Reflex Raman spectrometer (Renishaw, New Mills, UK), in a backscatter configuration. A diode-pumped laser (DPSS) with a 532 nm line was applied as excitation. Complementary measurements with a 785 nm diode-type laser line excluded resonance effects. Temperature investigations made using a Linkam DSC600 temperature cell cooled with liquid nitrogen under an argon atmosphere proved the structural stability of all samples in the temperature range of 80 K to 420 K.

### 2.5. Optical Properties and Models

The optical properties of all the investigated titanium nitride samples are expressed in terms of the complex dielectric function $\varepsilon =\varepsilon_{1}+i\varepsilon_{2}$, which, apart from the refractive n and extinction k indices, is one of the possibilities used to describe the interaction between light and matter. A material can exhibit plasmonic behavior when the real part of the dielectric function is negative $\varepsilon_{1}<0$ or equivalently n<k. In our work, the dielectric function was determined based on the results provided by Variable Angle Spectroscopic Ellipsometry (VASE). The raw experimental data produced by VASE are the *Ψ*(*λ*) and *Δ*(*λ*) values, which were obtained using two Woolam ellipsometers (RC2 and IR-VASE Mark II) for five angles of incidence θ: 55 deg, 60 deg, 65 deg, 70 deg, and 75 deg, in two spectral ranges: one of 193 nm to 1.69 μm and the second of 1.7 μm to 30 μm. The transition from *Ψ*(*λ*) and *Δ*(*λ*) to *ε*(*λ*) is not straightforward. It requires some assumptions—typically on the optical model of the sample and on the parametrized dispersion model of the dielectric function that describes the investigated material. In this approach, parameters of the dispersion model are variables, which values are optimized to minimize the difference between the raw ellipsometric parameters *Ψ*(*λ*) and *Δ*(*λ*) and their counterparts calculated based on the optical model and parametrized dielectric function. The main disadvantage of the above approach is that the dielectric function has to be postulated a priori and that what is fitted is the *Ψ*(*λ*) and *Δ*(*λ*) values, which do not have an intuitive meaning. Thus, it could be complicated to assess if differences between raw and modeled *Ψ*(*λ*) and *Δ*(*λ*) values have physical meaning, and if yes, what. In this work, using only an optical model, we calculate the dielectric function (we name it extracted). We note that we do not require a priori knowledge of the dispersion model that describes TiN*_x_* films. We are aware that we are neglecting the surface roughness or oxidation [29]. Still, on the other hand, such an approach allows us to more freely analyze the *ε*(*λ*) function, observe minor features, and more consciously interpret the deviation of the assumed dispersion model from experimental data.

The optical model of the TiN*_x_* samples depends on the spectral range. In the first spectral range of 193 nm to 1.7 μm, we assumed that the optical model for all TiN*_x_* samples deposited on a silicon substrate is just semi-infinite TiN*_x_* film. It is because, for the expected values of ε and thicknesses determined from Scanning Electron Microscopy (SEM) cross-section images, all samples are opaque. Formally, we used the following identities:

(1) $$\tan\Psi \cdot e^{i\Delta }=\frac{r_{p}}{r_{s}},\;r_{p}=\frac{\tan\left(\theta_{i}-\theta_{t}\right)}{\tan\left(\theta_{i}+\theta_{t}\right)},\;r_{s}=\frac{\sin\left(\theta_{t}-\theta_{i}\right)}{\sin\left(\theta_{t}+\theta_{i}\right)},$$

where $r_{s,p}$—Fresnel reflection coefficient for s,p polarization, $\theta_{i}$—angle of incidence (real), $\theta_{t}$—angle of refraction (complex), $n_{i}$—refractive index of air, $n_{t}$—refractive index of the investigated film (complex). Since Ψ, Δ, and $\theta_{i}$ are known, one can easily find $\theta_{t}$, and thus firstly n and k, and secondly $\varepsilon_{1}$ and $\varepsilon_{2}$. We also note that the proposed treatment is in agreement with the well-known expression for the “pseudo dielectric function”:

(2) $$\varepsilon =\sin^{2}\theta_{i}\left(1+\tan^{2}\theta_{i}\left(\frac{1-\rho }{1+\rho }\right)\right),$$

where $\rho =r_{p}/r_{s}$.

In the second spectral range of 1.7 μm to 30 μm, samples start to be transparent at the longer wavelengths, so the inclusion of substrate is necessary. In this case, all samples are treated as thin TiN*_x_* films of finite thickness on a silicon semi-infinite substrate. The silicon substrate is treated as semi-infinite because it is polished on one side, and the second side is very rough, which cancels all the reflections. Formally, we change the expressions for $r_{s,p}$ to be:

(3) $$r_{p}=\frac{\frac{\tan\left(\theta_{i}-\theta_{t}\right)}{\tan\left(\theta_{i}+\theta_{t}\right)}+A^{2}\frac{\tan\left(\theta_{t}-\theta_{\mathrm{Si}}\right)}{\tan\left(\theta_{t}+\theta_{\mathrm{Si}}\right)}}{1+A^{2}\frac{\tan\left(\theta_{i}-\theta_{t}\right)}{\tan\left(\theta_{i}+\theta_{t}\right)}\frac{\tan\left(\theta_{t}-\theta_{\mathrm{Si}}\right)}{\tan\left(\theta_{t}+\theta_{\mathrm{Si}}\right)}},\;r_{s}=\frac{\frac{\sin\left(\theta_{t}-\theta_{i}\right)}{\sin\left(\theta_{t}+\theta_{i}\right)}+A^{2}\frac{\sin\left(\theta_{\mathrm{Si}}-\theta_{t}\right)}{\sin\left(\theta_{\mathrm{Si}}+\theta_{t}\right)}}{1+A^{2}\frac{\sin\left(\theta_{t}-\theta_{i}\right)}{\sin\left(\theta_{t}+\theta_{i}\right)}\frac{\sin\left(\theta_{\mathrm{Si}}-\theta_{t}\right)}{\sin\left(\theta_{\mathrm{Si}}+\theta_{t}\right)}},$$

where $A=e^{i\left(n+ik\right)k_{0}t}$, $k_{0}=\frac{2\pi }{\lambda }$, t is the thin TiN*_x_* film thickness value, and $n_{\mathrm{Si}}$ is the silicon substrate refractive index value calculated from the measured Ψ and Δ values using Equation (1).

### 2.6. Ab Initio Calculations

All the calculations were performed within density functional theory (DFT) as implemented in the Vienna Ab Initio Simulation Package (VASP) code [39,40,41,42] with Projector augmented wave pseudopotentials [43,44] and Perdew-Burke-Ernzerhof (PBE) parametrization of the Generalized Gradient Approximation (GGA) functional [45]. For the sampling of the Brillouin zone, a dense 30 × 30 × 30 grid was used, while the plane wave energy cutoff was set to 500 eV. All the structures were optimized until the forces exerted on atoms were smaller than 10^−5^ eV/Å. Phonon spectrum was calculated using the Parlinski-Li-Kawazoe method supercell approach with the finite displacement method [46] as implemented in the Phonopy code [47]. Optical properties were calculated on the Random-Phase Approximation (RPA) level [48] as implemented in the VASP code [49].

## 3. Results

### 3.1. Structural Properties

Structural properties were investigated using Scanning Electron Microscopy (SU-70, Hitachi, Tokyo, Japan), Atomic Force Microscopy (AFM, Dimension Icon, Bruker, Bilerica, MA, USA), Secondary Ion Mass Spectroscopy, Grazing Incidence X-Ray Diffraction (GIXRD), Energy-dispersive X-ray spectroscopy (EDS, UltraDry silicon drift X-ray detector and Noran System 7 X-ray Microanalysis System, ThermoFisher Scientific, Waltham, MA, USA), and Raman spectroscopy techniques. The films’ thicknesses were estimated from the SEM images of samples cross-sections. An exemplary image of the cross-section of the stoichiometric sample is shown in Figure 1b. The exact values of thicknesses of all samples are shown in Table 1. As can be seen, the film thickness increases with a decrease in the nitrogen content, it is the lowest for the most nitrogen-rich sample, and it is the highest for the most nitrogen-deficient. Observed changes in thickness result partially from the decrease in nitrogen flux and increase in the argon flux. We note that we keep the total gas flux, consisting of argon and nitrogen fluxes, approximately constant in this experiment. Hence, a decrease in the nitrogen flux implies an increase in the argon flux. The latter one mainly determines the ejection rate of the titanium atoms from the target. Another explanation is the coverage of the titanium target by titanium nitride, which was found to decrease the deposition rate [50]. The analysis of the SEM images of the samples’ surfaces illustrates the increasing granularity of the surfaces, which is further confirmed by the AFM images’ analysis. The AFM results show that relatively low Root-Mean-Square (RMS) values characterize samples number 1 to 4, whereas significantly larger RMS values characterize samples number 5 to 7. The higher growth rate can partially explain the observed changes in the RMS values, but a change of the growth mechanism is also possible [51]. SEM images of cross-sections, SEM images of surfaces, and AFM images of surfaces of all samples are shown in Supporting Information, Figures S2–S22.

Results of the SIMS measurements are shown in Table 1. As can be seen, the composition changes from TiN_1.21±0.02_ for sample 1 to TiN_0.84±0.03_ for sample 7, with sample 5 being almost stoichiometric. Hence, there are four samples with excess nitrogen content and only two with deficient nitrogen content. This situation results from the different stability of the sputtering process for samples characterized by excess and deficient nitrogen content. A precise composition control is easily achievable for the first class of samples in our experimental setup since the sputtering process is stable. In contrast, the sputtering process is very susceptible to the process parameters for the samples with nitrogen deficiency, and thus, it is not easy to achieve the exact desired composition.

We also correlate the TiN*_x_* composition with its optical properties, namely the wavelength at which the real part of the dielectric function equals zero λ@ε_1_ = 0. It is a popular treatment for plasmonic TiN*_x_*. We found a linear dependence, as shown in Figure 1c. This result fits well with the trends reported in the literature [52,53]. Formally:

(4) $$x\;\left(\mathrm{composition}\right)=\left(0.00170\pm 0.00011\right)\cdot \;{\lambda |}_{\varepsilon_{1}=0}+\left(0.17\pm 0.06\right),$$

Uncertainties of the above linear dependence parameters are calculated by considering both the composition’s and wavelength’s uncertainties.

The results of the GIXRD measurements are shown in Figure 1d. Collected XRD patterns are typical for titanium nitride and can be indexed within space group $\mathrm{Fm}\bar{3}m$ (ICSD:183415) with no indication of secondary phases [54,55]. The presence of even five quite broad peaks in the XRD patterns proves that the samples are polycrystalline. The relative intensities of different peaks reflect changes in the preferred growth orientation but not in the crystal structure. Calculated lattice constants are given in Table 1. However, the interpretation of the influence of the composition that results from the process parameters on the values of *a*_0_ is demanding.

The conducted EDS studies provide information on the composition of the layers. The results showed that the layers, with an accuracy of sensitivity of the EDS technique, consist only of titanium and nitrogen (silicon signal comes from the substrate). However, the exact extraction of the nitrogen to titanium nitride ratio is difficult due to limited energy resolution and close nitrogen and titanium peak positions. A decrease in the silicon peak amplitude well correlates with the sample thickness.

### 3.2. Optical Properties in the UV-VIS Range

**Figure 2:**
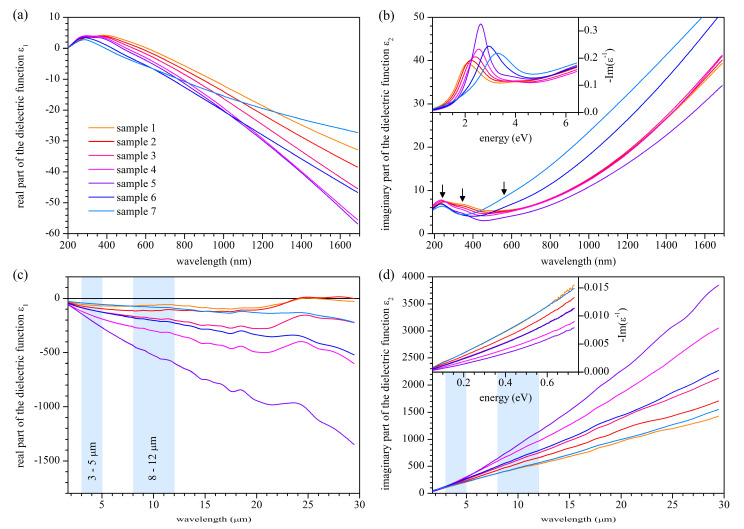
Extracted values of the real (**a**,**c**) and imaginary (**b**,**d**) part of the dielectric function in the 193 nm to 1.69 μm (**a**,**b**) or 1.7 μm to 30 μm (**c**,**d**) spectral range. Arrows in Figure (**b**) point to the optical transitions. Insets in Figures (**b**,**d**) show the loss function (minus the imaginary part of the reciprocal dielectric function) as a function of energy.

The results of the calculations of the extracted dielectric function are shown in Figure 2 (all the *Ψ*(*λ*) and *Δ*(*λ*) values, which are raw data, and which were used for calculation of the extracted dielectric function, which is shown in the Supporting Information, Figures S23–S30). In the visible and near-infrared range, the dielectric function behaves typically for metallic titanium nitride. In the range of 193 nm to 800 nm, in the real and imaginary part, a few optical transitions are visible at approximately 2.1–2.4 eV, 3.5–3.8 eV, 5.1–5.8 eV, and 6.4–6.8 eV, depending on stoichiometry (see Table 2). For wavelengths longer than 800 nm, only a strong Drude contribution can be seen. The real part of ε changes its sign from positive to negative in the range 392.5 nm to 608.0 nm, depending on stoichiometry (see Table 1). This latter feature is rendered in the loss function −Im(ε−1) as a peak that can be seen in the inset in Figure 2b. Concerning the real part of the dielectric function, the sample characterized by the most negative values is the stoichiometric one, and the less stoichiometric sample is the less negative $\varepsilon_{1}$. For all the samples, the $\varepsilon_{1}$ continuously decreases (except for the most energetic part of the spectra), and no sample loses its plasmonic properties when the wavelength increases. Regarding the imaginary part of the dielectric function, the least lossy sample is the stoichiometric one. The lesser the stoichiometric sample, the higher the losses, and it seems that samples characterized by the nitrogen-deficiency are lossier than those characterized by nitrogen excess. Optical measurements performed four months after the first ones do not reveal any significant changes in the optical properties, so the samples are quite stable. The plasmonic figure of merit value expressed as $\mathrm{FoM}=\left|\varepsilon_{1}\right|/\varepsilon_{2}$, in the best case of the stoichiometric sample, reaches the value of 1.86, which is an excellent result for a sputtering process conducted at room temperature [56].

Formally, the dielectric function ε in the range of 193 nm to 1.69 μm can be described analytically as a sum of one Drude component and four Lorentz oscillators:

(5) $$\varepsilon \left(E\right)=\varepsilon_{\infty }+\frac{E_{\mathrm{pu}}^{2}}{-E^{2}-i\;E\;\Gamma_{D}}+\sum_{i}\frac{f_{j}\;E_{j}^{2}}{E_{j}^{2}-E^{2}-i\;E\;\Gamma_{j}},$$

where $\varepsilon_{\infty }$ is the high-frequency dielectric constant, $E_{\mathrm{pu}}$ is the plasma energy in the Drude model, $\Gamma_{D}$ is the damping factor in the Drude model, $f_{j}$ is the strength, $E_{j}$ is the energy, and $\Gamma_{j}$ is the damping factor of the *j*-th Lorentz oscillator (*i* is the imaginary unit).

Values of all parameters that describe ε can be found in Table 2. As shown further, the energies of optical transitions correspond well to the TiN band structure and imaginary part of the dielectric function calculated using the *ab inito* method. However, we note that values in Table 2 should be treated with some care since the peaks related to optical transitions are not distinct or well separated in the extracted dielectric function. Thus, the fitted values are loaded with high uncertainty and characterized by low uniqueness. We also note that it was difficult to fit the assumed dispersion model accurately to the data related to sample 5 (the stoichiometric one).

### 3.3. Optical Properties in the Infrared Range

In the infrared range, the optical properties of all the samples are governed mainly by the Drude model, which manifests as negative and decreasing with increasing wavelength real part of the dielectric function and positive and increasing imaginary part of the dielectric function. The sample characterized by the most negative value of $\varepsilon_{1}$ is the stoichiometric one, and the less stoichiometric the sample is, the less negative $\varepsilon_{1}$ will be. In the case of the imaginary part of the dielectric function, contrary to the visible range, the lossiest sample is the stoichiometric one, and the less stoichiometric sample, the lower the losses. For wavelengths above 20 μm, for part of the samples, especially for the nitrogen-rich samples, a distinct increase in the value of $\varepsilon_{1}$ is observed. The origin of such behavior is unknown. However, a comparison of curves for samples 1 and 2 with a curve for sample 7 and a curve for sample 3 with a curve for sample 6 suggests a composition-dependent physical factor. Because of the interpretational doubt and relatively large scale of this feature, we will model the dielectric function only in the range up to 20 μm. Regarding minor features observed in the real part of the dielectric function for all the samples at approximately 11 μm, 15 μm, and 18 μm, we assume that they are of phonon origin. Contrary to the real part, the imaginary part of the dielectric function does not show any distinct features, which can result from the fact that even if there indeed occur some minor features, they have too little amplitude in comparison to $\varepsilon_{2}$ values to be revealed.

As previously said, the dielectric function in the infrared range up to 20 μm of all titanium nitride samples is governed mainly by the Drude model. However, to be precise, an additional Lorentz oscillator centered at zero energy has to be included for part of the samples to render the experimental values accurately. Formally,

(6) $$\varepsilon \left(E\right)=\varepsilon_{\infty }+\frac{E_{\mathrm{pu}}^{2}}{-E^{2}-i\;E\;\Gamma_{D}}+\frac{f_{0}E_{0}^{2}}{-E^{2}-i\;E\;\Gamma_{0}},$$

where $f_{0}E_{0}^{2}$ and $\Gamma_{0}$ are the parameters of the additional Lorentz oscillator. We note that since $E_{0}\;$= 0 we treat $f_{0}E_{0}^{2}$ as one non-zero parameter. We are not able at this moment to determine the origin of this oscillator; particularly, we cannot determine if it is technology-dependent (specific sputtering conditions, properties of surface) or a feature related to the investigated material. The ab initio calculations will provide a possible explanation; however, it would be rather a suggestion than proof. We also note that this additional term resembles the Drude term and could be named the second Drude term. However, in such a case, it will suggest a correlation with an additional Drude term required in some cases to describe an anisotropic system. Because we do not have any premises that our films are anisotropic, but we have some premises that there could be very low energy optical transitions in titanium nitride samples, we name the additional term as Lorentz oscillator. We also note that we excluded that the observed deviation from the Drude model is of the instrumental origin by performing an additional experiment. We evaporated 300 nm thick silver film on a glass substrate and found the dielectric function. In the range of 800 nm to 20 μm, no deviation from the Drude model was observed (see Supporting Information, Figure S31).

The model presented here is valid from 800 nm up to 20 μm with parameters shown in Table 3. Contrary to data in Table 2 describing optical properties of TiN*_x_* samples in the visible range, data in Table 3 are much more reliable and also pretty unique. Pictures illustrating experimental and simulated values of the dielectric function in the considered range are shown in Supporting Information Figures S32–S38.

### 3.4. Ab Initio Calculations

**Figure 3:**
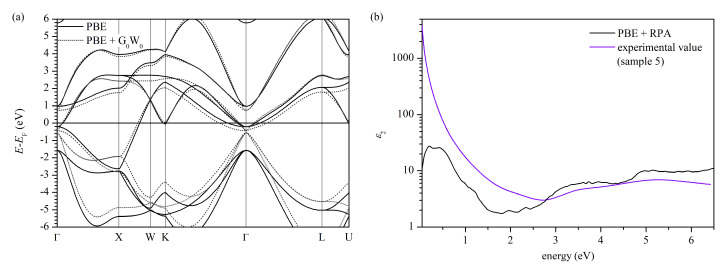
(**a**) Band structure and (**b**) imaginary part of the dielectric function.

The results of the ab initio calculations, i.e., the electronic band structure and the imaginary part of the dielectric function of unperturbed stoichiometric titanium nitride, are presented in Figure 3. The analysis of the band structure clearly shows that the simulated system is metallic. The Fermi level crosses the band structure near the Γ, K, and U points and inside the X-W path. The bottom of the “conduction-like” band, located at Γ point at approximately −0.21 eV, and the top of the “valence-like” band, located at Γ at approximately −1.57 eV, are triply degenerated, which is revealed as a band splitting on Γ-K direction. The band splitting that forms parallel bands on Γ-K and Γ-K direction is essential for further interpretation. We note that our results agree with previous theoretical studies [57,58,59,60,61,62,63] and photoemission spectroscopy studies, which reveal a high density of states just below the Fermi level [64,65]. The many-body G_0_W_0_ correction (one-shot GW correction) to quasiparticle energies significantly shifts the band positions, especially those below the Fermi level. The most striking effect is decreased separation energy between the “valence-like” and “conduction-like” bands at the center of the Brillouin zone, whereas the bands’ shapes seem less affected. In Figure 3b, one can find the optical properties, namely the imaginary part of the dielectric function, which were calculated using random phase approximation (RPA). The PBE + RPA results seem to agree with previous work [62] and our experimental dielectric function for the stoichiometric sample (please see the peak position in Figure 3b at 1.9 eV, 3.7 eV, 5.2 eV, and 6.4 eV, and values in Table 2). The main difference between the simulated and experimental dielectric function is that in the experimental curve, the peaks are more blurred compared to quite distinct peaks in the simulated curve. A possible explanation is that our sample is polycrystalline; thus, experimental results are obtained from a set of many grains. Suppose the optical properties (i.e., parameters of the Lorentz oscillators describing each grain) differ within the set of grains. In that case, the resulting optical properties will be a convolution of all particular optical properties with weight proportional to their contributions. As a consequence, all the peaks will blur.

The most characteristic feature of the imaginary part of the simulated dielectric function is the existence of low-energy peaks below 1 eV (at 0.16 eV, 0.41 eV, and 0.87 eV in Figure 3b). We note that this peak is not related to the Drude component since our calculations include only the interband transitions, and the Drude component describes the intraband transitions, or in other words, the free-electron contribution. According to the calculated band structure in Figure 3a, such low-energy transitions are possible in the vicinity of the center of the Brillouin zone between almost parallel bands. The parallel-band effects in interband optical absorption are a known effect [66,67,68], found experimentally, e.g., in aluminum [69,70], or gold and platinum alloys [71,72]. Moreover, the band splitting leading to parallel band absorption is seen in all cited works related to band structure calculations. The low-energy optical transitions are also rendered in the optical conductivity calculations [59]. Thus, there are many theoretical/simulational premises in which, apart from the Drude component, a Lorentz oscillator has to be included to describe the dielectric function accurately in the spectral range of 800 nm to 20 μm. However, one should remember that these all are still only suggestions, not formal proof.

### 3.5. Raman Measurements

**Figure 4:**
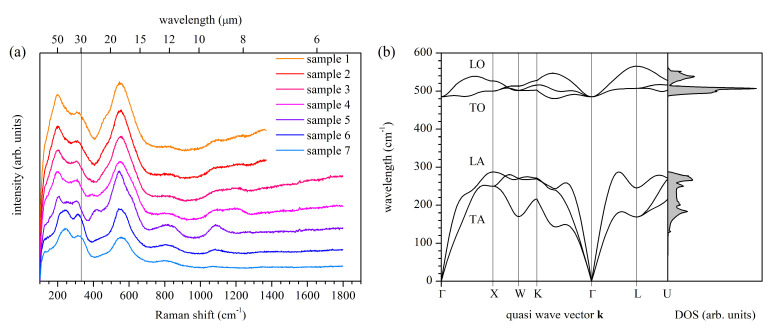
(**a**) Experimental Raman spectra of all samples and (**b**) phonon band structure with a corresponding density of states.

Results of the Raman measurements on all samples are shown in Figure 4a. All the spectra are typical for metallic TiN*_x_*, despite some differences from published reports [73,74,75,76,77] in peak number and positions, which, however, can be very technology-dependent. The main features of the Raman spectra are peaks related to both acoustic and optical phonons, both first and second-order, which for the stoichiometric sample are located at 200 cm^−1^, 255 cm^−1^, 304 cm^−1^, 407 cm^−1^, 540 cm^−1^, 551 cm^−1^, 804 cm^−1^, and 1090 cm^−1^. We note that, typically, only optical phonons from the center of the Brillouin zone are observed in Raman spectra. The occurrence of the first-order acoustic phonons means that the selection rules for the Raman scattering process are violated, which can happen, for example, in the presence of structural defects, which also include the grain boundaries. On the other hand, the first-order acoustic modes are also observed in epitaxial layers characterized by the low defect concentration [75], suggesting that the violation of the typical Raman selection rules can be an intrinsic property of titanium nitride. Contrary to other authors, we do not dare to assign observed peaks to corresponding phonons since our experiments and theoretical calculations do not provide enough information to make reliable assignments. To show the complexity of this task, we use the results of our ab initio calculations, as shown in Figure 4b. We note that our calculations agree with the inelastic phonon scattering experiment [78] and previously reported calculations [79]. Considering two peaks located at 540 cm^−1^ and 551 cm^−1^, one can suspect that these peaks are optical modes that reflect the phonon density of states at the Γ-X and Γ-K direction related to the LO branch. However, our samples are polycrystalline and sputtered on a mismatched substrate. Thus, our samples can be affected by unidentified stress, which is known to seriously modify the phonons’ properties [77], e.g., by stiffening the lattice vibrations. Consequently, it may turn out that the 540 cm^−1^ and 551 cm^−1^ peaks are related to the TO phonon branch, which is shifted to higher energies due to stress. Moreover, because the second-order features are observed in the Raman spectra (undoubtedly, the peaks at 804 cm^−1^ and 1090 cm^−1^ are second-order features), it is possible that the second-order acoustic modes also contribute to the Raman spectra in the range of 500 cm^−1^ to 600 cm^−1^, which makes the mode identification even more challenging.

Conclusions that can be drawn from the Raman measurements are two. The first one is that despite changes in composition, all the samples have similar Raman spectra, and thus, the structural properties that influence phonon properties are also similar. In particular, observed small changes in the Raman spectra could not be related to phase transition or change of the rock-salt crystal structure characterizing our TiN*_x_* samples. The second conclusion is that the Raman spectra are rich. Assuming that light absorption by phonons in the infrared range will be at least somehow similar to inelastic light scattering by phonons in the visible range, one can expect that dielectric function can be affected by phonons, up to the wavelength of 8 μm. In Figure 4a, an additional scale at the top of the plot expresses the phonon energy in the wavelength units to help compare Figure 2 with Figure 4. Translating the energy range in which the density of optical phonons is non-zero, i.e., approximately from 485 cm^−1^ to 566 cm^−1^ according to Figure 4b, into the wavelength range, we obtain approximately the range of 17.5 μm to 20.4 μm. Particularly, the Raman peak at 551 cm^−1^ can be related to the feature in the real part of the dielectric function at 18 μm. Other features observed in $\varepsilon_{1}$, if of phonon origin, should arise from a multi-phonon absorption process.

## 4. Conclusions

The main scientific results of our work are experimental dielectric function values obtained using Variable Angle Spectroscopic Ellipsometry in the spectral range of 193 nm to 30 μm. We demonstrate that thin titanium nitride TiN*_x_* films can exhibit plasmonic properties continuously from 400 nm up to 30 μm for stoichiometry in the range of *x* = 1.21 ± 0.02 to *x* = 0.84 ± 0.03, and we provide reliable parametrized analytical models valid in the range of 800 nm to 20 μm. For wavelengths longer than 800 nm, we found that the optical properties of TiN*_x_* generally follow the Drude model. However, we identified an apparent deviation from the Drude model for part of the samples. An additional Lorentz term can describe this deviation, but its origin is still unknown at the moment of writing this publication. Since it is the first report on the dielectric function for such long wavelengths, we could not support our consideration with the literature, limiting ourselves to providing possible explanations. The first pending question is whether the observed deviation is a technology-specific issue, such as the grain boundaries’ influence limiting the electron movement or material property. If it is a material property, the next question is if the considered deviation is related to the band structure, like the parallel-band effect, or maybe some corrections to the Drude model are required, similar to those applied for electrically conducting polymers [80].

## Figures and Tables

**Table 1:** Measured values of selected parameters that characterize deposited thin films.

#	d (nm)	RMS (nm)	λ@ε_1_ = 0 (nm)	*x* in TiN*_x_* −	*a*_0_ (nm)
1	151	1.02	608.0 ± 0.5	1.21 ± 0.02	0.4260
2	169	0.99	572.5 ± 0.5	1.16 ± 0.02	0.4255
3	188	0.99	537.0 ± 0.5	1.10 ± 0.01	0.4263
4	230	0.96	501.0 ± 0.5	1.04 ± 0.01	0.4280
5	415	2.42	479.0 ± 0.5	0.99 ± 0.01	0.4234
6	515	2.43	428.5 ± 0.5	0.93 ± 0.02	0.4230
7	542	2.46	392.5 ± 0.5	0.84 ± 0.03	0.4220

**Table 2:** Values of all parameters that describe the dielectric function ε of all the samples in the range 193 nm to 1.69 μm.

	Sample 1	Sample 2	Sample 3	Sample 4	Sample 5	Sample 6	Sample 7
$E_{\mathrm{pu}}$ (eV)	6.61	6.73	6.80	6.82	6.30	7.42	8.22
$\Gamma_{D}$ (eV)	0.66	0.63	0.61	0.57	0.71	0.91	1.30
*f*_1_	0.16	0.07	0.81	1.07	0.15	0.45	0.64
$E_{1}$ (eV)	2.39	2.30	2.30	2.15	2.16	2.17	2.22
$\Gamma_{1}$ (eV)	0.93	0.70	1.97	1.90	0.68	1.09	1.41
*f*_2_	0.63	0.26	0.54	0.56	0.56	0.28	-
$E_{2}$ (eV)	3.49	3.51	3.60	3.67	3.68	3.85	-
$\Gamma_{2}$ (eV)	1.49	1.07	1.36	1.34	1.20	1.63	-
*f*_3_	5.53	6.40	2.99	2.61	2.12	2.44	1.62
$E_{3}$ (eV)	5.54	5.84	5.19	5.20	5.16	5.34	5.07
$\Gamma_{3}$ (eV)	4.73	5.44	3.27	2.86	2.41	2.85	2.62
*f*_4_	0.31	0.04	2.03	1.89	1.50	1.42	2.00
$E_{4}$ (eV)	6.68	6.47	6.76	6.56	6.43	6.76	6.63
$\Gamma_{4}$ (eV)	1.70	0.72	3.68	3.01	2.43	2.88	3.57

**Table 3:** Values of all parameters that describe the dielectric function ε for all the samples in the range of 800 nm–20 μm.

	Sample 1	Sample 2	Sample 3	Sample 4	Sample 5	Sample 6	Sample 7
$E_{\mathrm{pu}}$ (eV)	6.45	6.40	6.57	6.70	6.10	7.13	8.10
$\Gamma_{D}$ (eV)	0.71	0.57	0.58	0.47	0.35	0.68	1.19
$f_{0}E_{0}^{2}$ (eV)	-	-	1.32	2.41	4.97	2.07	0.93
$\Gamma_{0}$ (eV)	-	-	0.065	0.065	0.061	0.070	0.075

## Data Availability

Not applicable.

## Supplementary Materials

The following are available online at https://www.mdpi.com/article/10.3390/ma14227095/s1. Figure S1. XRD patterns of sample number 5 (the most stoichiometric one) collected for the incidence angle values in the range of 0.2° to 2.5°. Patterns collected at the high incidence angles (*α*_i_ > 1.0°) show diffraction peaks from the Si substrate. The highest signal-to-noise ratio is observed for *α*_i_ = 0.4°; Figure S2. SEM image of a cross-section of sample number 1; Figure S3. SEM image of a cross-section of sample number 2; Figure S4. SEM image of a cross-section of sample number 3; Figure S5. SEM image of a cross-section of sample number 4; Figure S6. SEM image of a cross-section of sample number 5; Figure S7. SEM image of a cross-section of sample number 6; Figure S8. SEM image of a cross-section of sample number 7; Figure S9. SEM image of a surface of sample number 1; Figure S10. SEM image of sample number 2; Figure S11. SEM image of sample number 3; Figure S12. SEM image of sample number 4; Figure S13. SEM image of sample number 5; Figure S14. SEM image of sample number 6; Figure S15. SEM image of sample number 7; Figure S16. AFM image of sample number 1; Figure S17. AFM image of sample number 2; Figure S18. AFM image of sample number 3; Figure S19. AFM image of sample number 4; Figure S20. AFM image of sample number 5; Figure S21. AFM image of sample number 6; Figure S22. AFM image of sample number 7; Figure S23. Ellipsometric parameters Ψ and Δ as a function of wavelength in two spectral ranges for sample number 1; Figure S24. Ellipsometric parameters Ψ and Δ as a function of wavelength in two spectral ranges for sample number 2; Figure S25. Ellipsometric parameters Ψ and Δ as a function of wavelength in two spectral ranges for sample number 3; Figure S26. Ellipsometric parameters Ψ and Δ as a function of wavelength in two spectral ranges for sample number 4; Figure S27. Ellipsometric parameters Ψ and Δ as a function of wavelength in two spectral ranges for sample number 5; Figure S28. Ellipsometric parameters Ψ and Δ as a function of wavelength in two spectral ranges for sample number 6; Figure S29. Ellipsometric parameters Ψ and Δ as a function of wavelength in two spectral ranges for sample number 7; Figure S30. Ellipsometric parameters Ψ and Δ as a function of wavelength in two spectral ranges for Si substrate; Figure S31. Extracted and simulated dielectric function in the low-energy range for freshly evaporated 300 nm thick Ag film on glass; Figure S32. Extracted and simulated dielectric function in the low-energy range for sample number 1; Figure S33. Extracted and simulated dielectric function in the low-energy range for sample number 2; Figure S34. Extracted and simulated dielectric function in the low-energy range for sample number 3; Figure S35. Extracted and simulated dielectric function in the low-energy range for sample number 4; Figure S36. Extracted and simulated dielectric function in the low-energy range for sample number 5; Figure S37. Extracted and simulated dielectric function in the low-energy range for sample number 6; Figure S38. Extracted and simulated dielectric function in the low-energy range for sample number 7.

## Abbreviations

AFM	Atomic Force Microscopy
CMOS	Complementary Metal-Oxide-Semiconductor
DFT	Density Functional Theory
EDS	Energy-dispersive X-ray spectroscopy
FTIR	Fourier transform infrared (spectroscopy)
GGA	Generalized Gradient Approximation
GIXRD	Grazing Incidence X-ray Diffraction
LO	Longitudinal Optical (phonon)
PBE	Perdew-Burke-Ernzerhof (parametrization)
RMS	Root-Mean-Square
RPA	Random-Phase Approximation
SEM	Scanning Electron Microscopy
SERS	Surface-Enhanced Raman Spectroscopy
SIMS	Secondary Ion Mass Spectroscopy
TO	Transverse Optical (phonon)
UV	Ultra-violet (spectral range)
VASE	Variable-Angle Spectroscopic Ellipsometry
VIS	Visible (spectral range)
