# Supplementary material for: Titanium Nitride as a Plasmonic Material from Near-Ultraviolet to Very-Long-Wavelength Infrared Range
Source: Materials (Basel). 2021 Nov 22;14(22):7095. doi: 10.3390/ma14227095 (PMC8622232; doi:10.3390/ma14227095)
Supplement: Supplementary file 1 [file materials-14-07095-s001.zip › materials-1434056-supplementary.pdf]

materials

Supplementary Materials

Titanium Nitride as a Plasmonic Material from Near-Ultraviolet to Very-Long-Wavelength Infrared Range

Jarosław Judek [1,*], Piotr Wróbel [2], Paweł Piotr Michałowski [3], Monika Ożga [4], Bartłomiej Witkowski [4], Aleksandra Seweryn [4], Michał Struzik [5], Cezariusz Jastrzębski [5] and Krzysztof Zberecki [5]

1 Institute of Microelectronics and Optoelectronics, Warsaw University of Technology, Koszykowa 75, 00-662 Warsaw, Poland
2 Faculty of Physics, University of Warsaw, Pasteura 5, 02-093 Warsaw, Poland; piotr.wrobel@fuw.edu.pl
3 Łukasiewicz Research Network—Institute of Microelectronics and Photonics, Aleja Lotnikow 32/46, 02-668 Warsaw, Poland; pawel.michalowski@imif.lukasiewicz.gov.pl
4 Institute of Physics, Polish Academy of Sciences, Aleja Lotników 32/46, 02-668 Warsaw, Poland; ozga@ifpan.edu.pl (M.O.); bwitkow@ifpan.edu.pl (B.W.); aseweryn@ifpan.edu.pl (A.S.)
5 Faculty of Physics, Warsaw University of Technology, Koszykowa 75, 00-662 Warsaw, Poland; michal.struzik@pw.edu.pl (M.S.); cezariusz.jastrzebski@pw.edu.pl (C.J.); krzysztof.zberecki@pw.edu.pl (K.Z.)
* Correspondence: jaroslaw.judek@pw.edu.pl

Citation: Judek, J.; Wróbel, P.; Michałowski, P.P.; Ożga, M.; Witkowski, B.; Seweryn, A.; Struzik, M.; Jastrzębski, C.; Zberecki, K. Titanium Nitride as a Plasmonic Material from Near-Ultraviolet to Very-Long-Wavelength Infrared Range. *Materials* **2021**, *14*, 7095. https://doi.org/10.3390/ma14227095

Academic Editor: Alexander N. Obraztsov

Received: 8 October 2021
Accepted: 17 November 2021
Published: 22 November 2021

Publisher's Note: MDPI stays neutral with regard to jurisdictional claims in published maps and institutional affiliations.

Copyright: © 2021 by the authors. Licensee MDPI, Basel, Switzerland. This article is an open access article distributed under the terms and conditions of the Creative Commons Attribution (CC BY) license (https://creativecommons.org/licenses/by/4.0/).

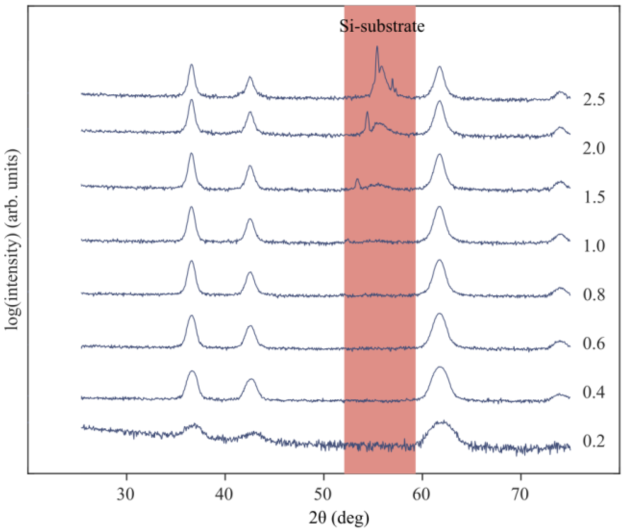

Figure S1. XRD patterns of sample number 5 (the most stoichiometric one) collected for the incidence angle values in the range of 0.2º to 2.5º. Patterns collected at the high incidence angles ($\alpha_i > 1.0º$) show diffraction peaks from the Si substrate. The highest signal-to-noise ratio is observed for $\alpha_i = 0.4º$.

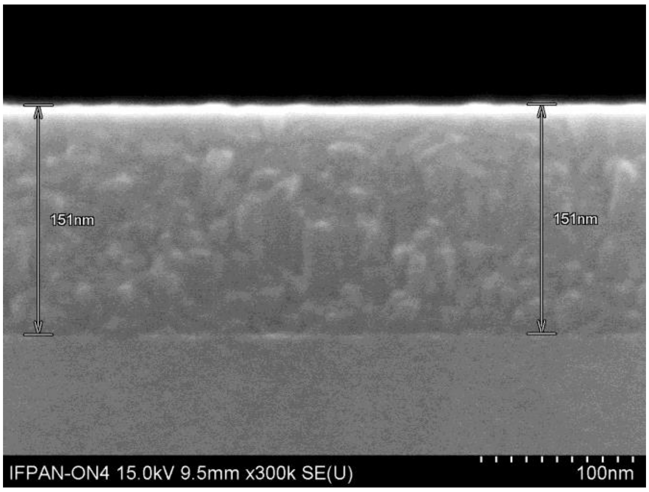

Figure S2. SEM image of a cross-section of sample number 1.

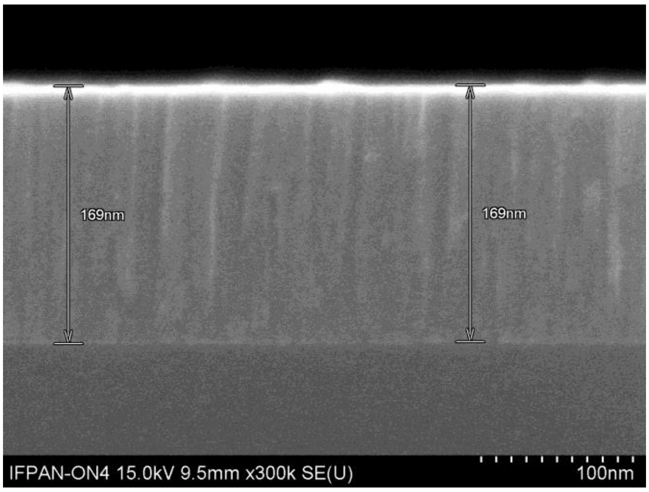

Figure S3. SEM image of a cross-section of sample number 2.

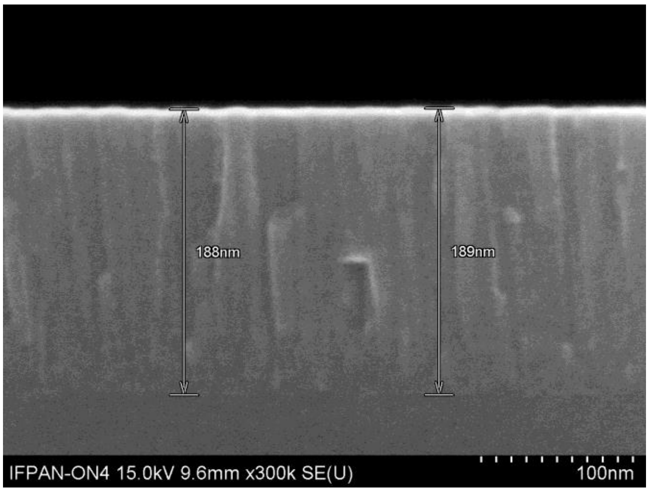

Figure S4. SEM image of a cross-section of sample number 3.

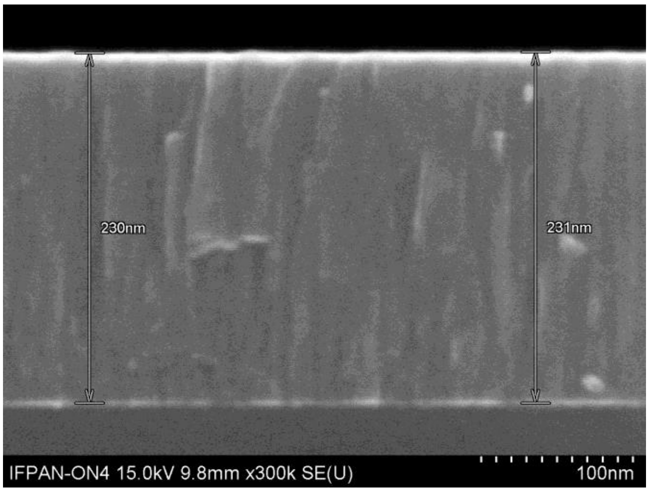

Figure S5. SEM image of a cross-section of sample number 4.

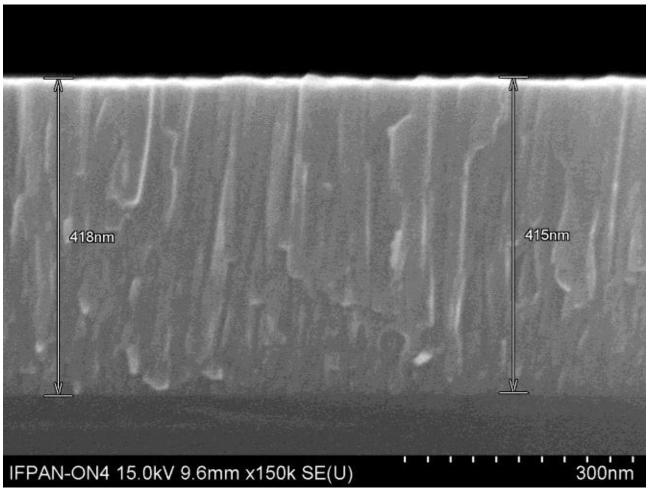

Figure S6. SEM image of a cross-section of sample number 5.

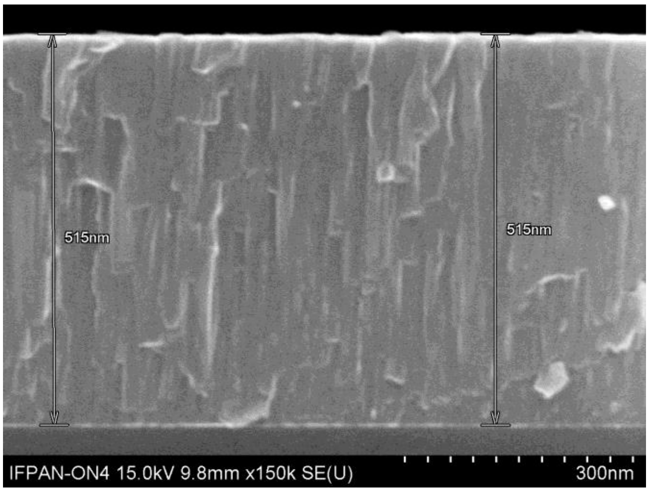

Figure S7. SEM image of a cross-section of sample number 6.

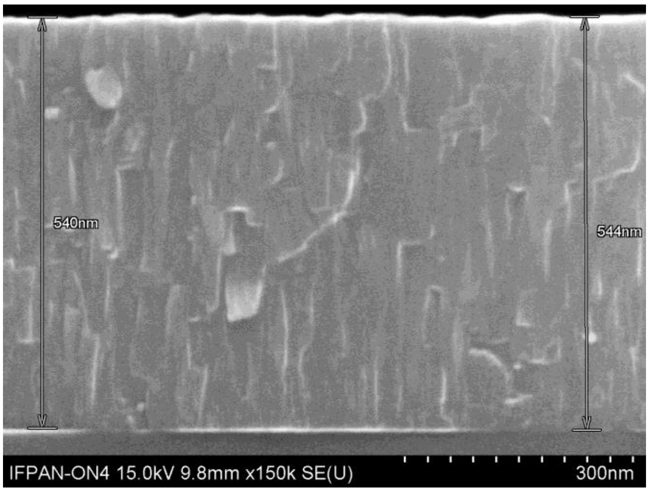

Figure S8. SEM image of a cross-section of sample number 7.

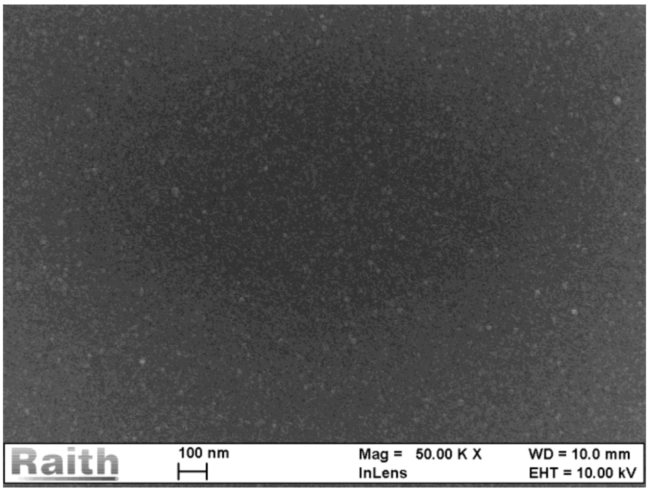

Figure S9. SEM image of a surface of sample number 1.

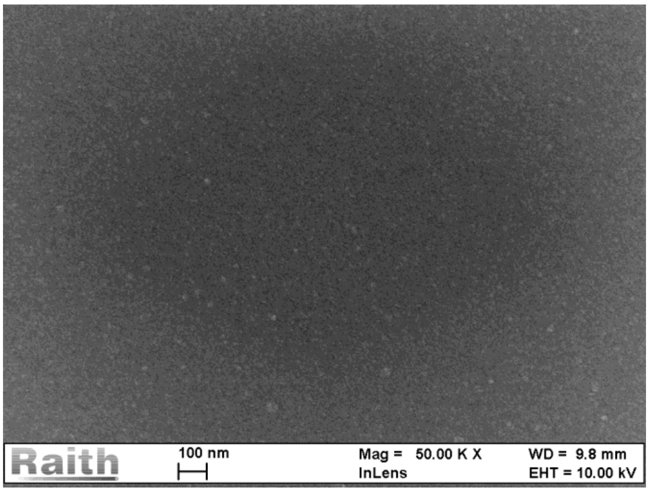

Figure S10. SEM image of sample number 2.

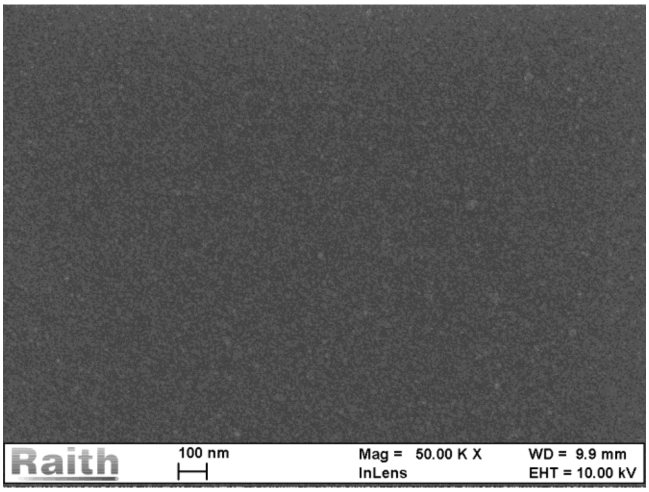

Figure S11. SEM image of sample number 3.

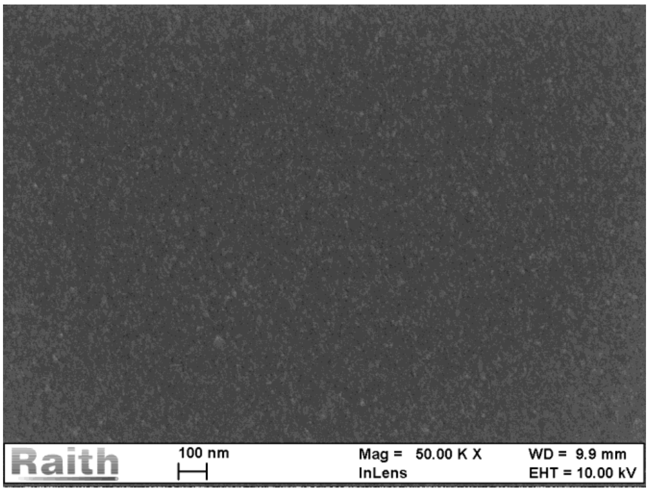

Figure S12. SEM image of sample number 4.

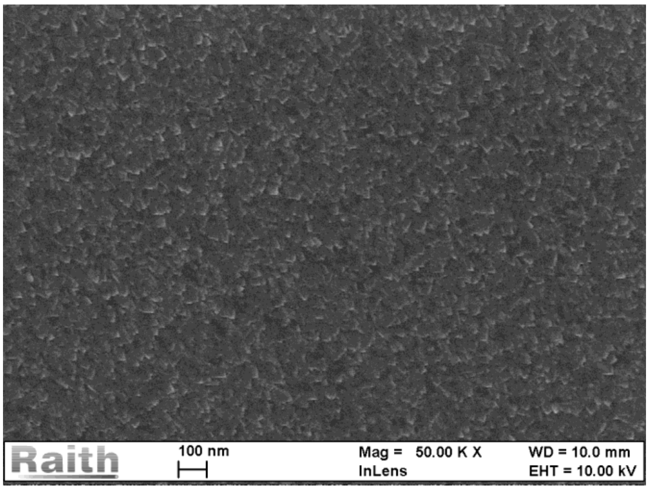

Figure S13. SEM image of sample number 5.

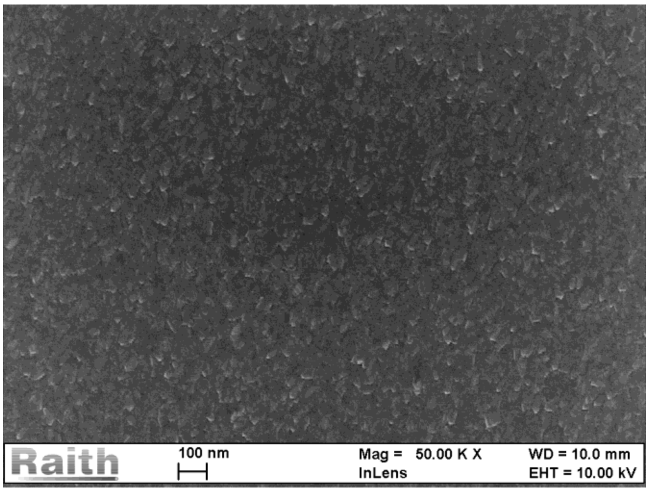

Figure S14. SEM image of sample number 6.

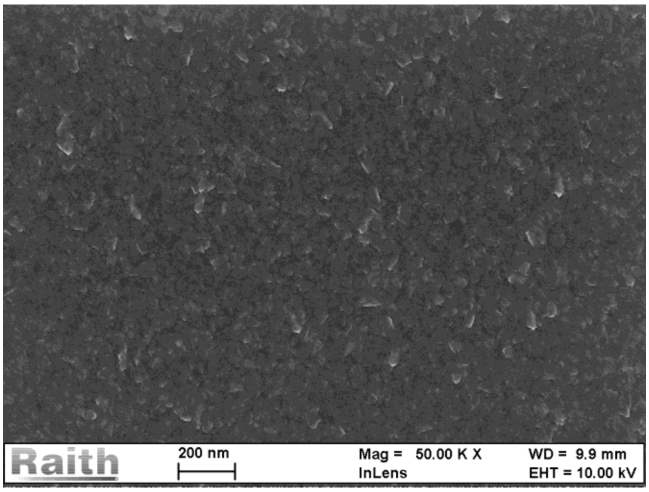

Figure S15. SEM image of sample number 7.

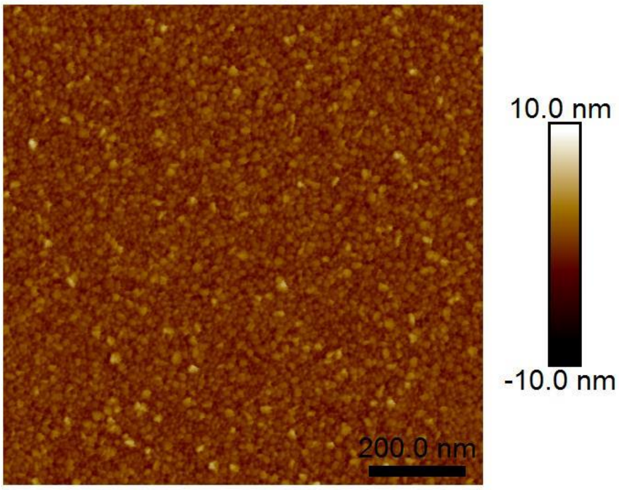

Figure S16. AFM image of sample number 1.

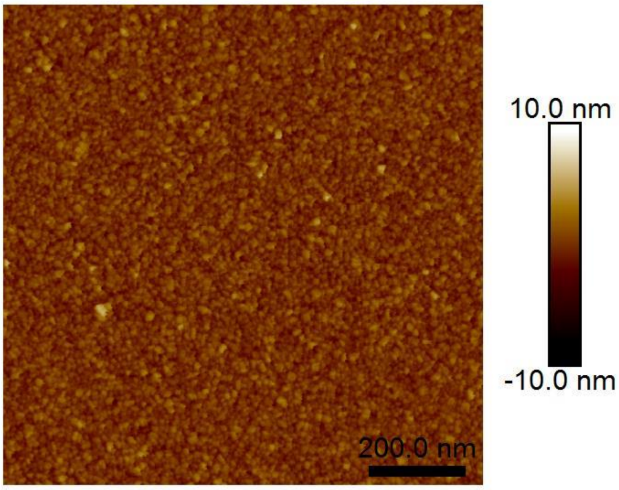

Figure S17. AFM image of sample number 2.

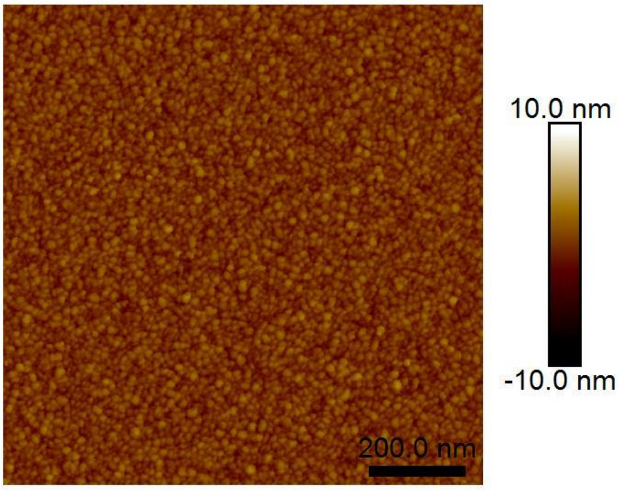

Figure S18. AFM image of sample number 3.

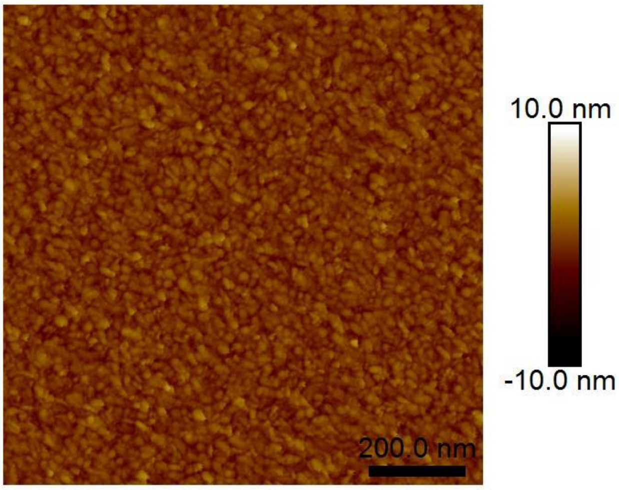

Figure S19. AFM image of sample number 4.

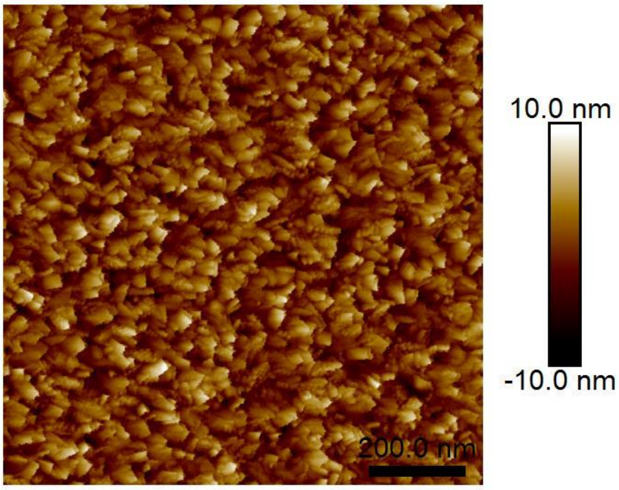

Figure S20. AFM image of sample number 5.

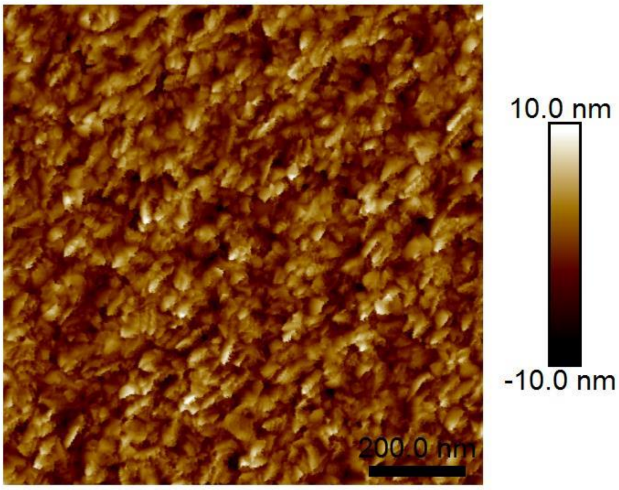

Figure S21. AFM image of sample number 6.

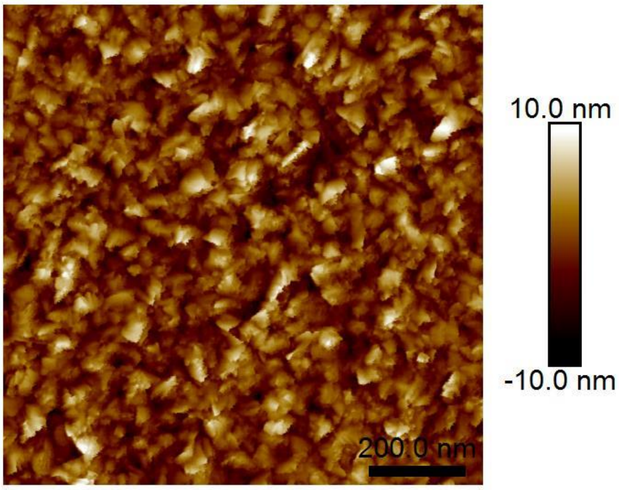

Figure S22. AFM image of sample number 7.

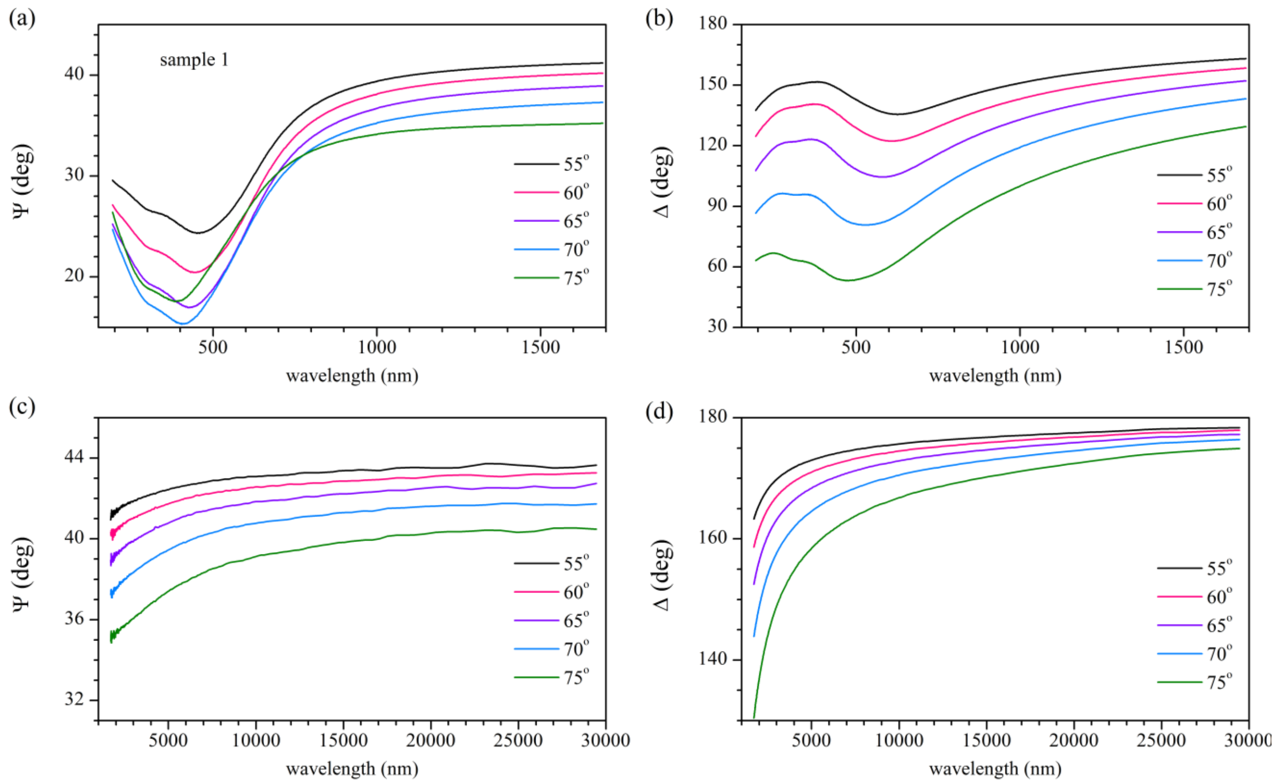

Figure S23. Ellipsometric parameters Ψ and Δ as a function of wavelength in two spectral ranges for sample number 1.

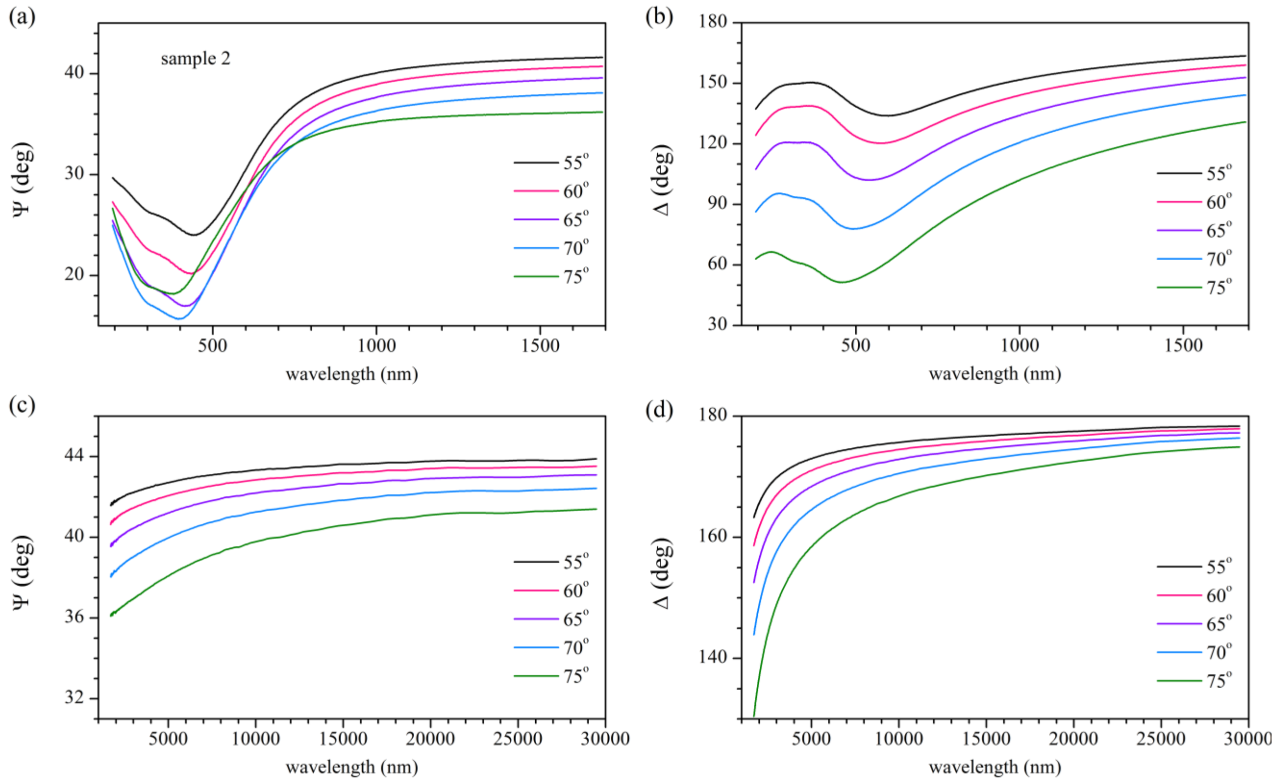

Figure S24. Ellipsometric parameters Ψ and Δ as a function of wavelength in two spectral ranges for sample number 2.

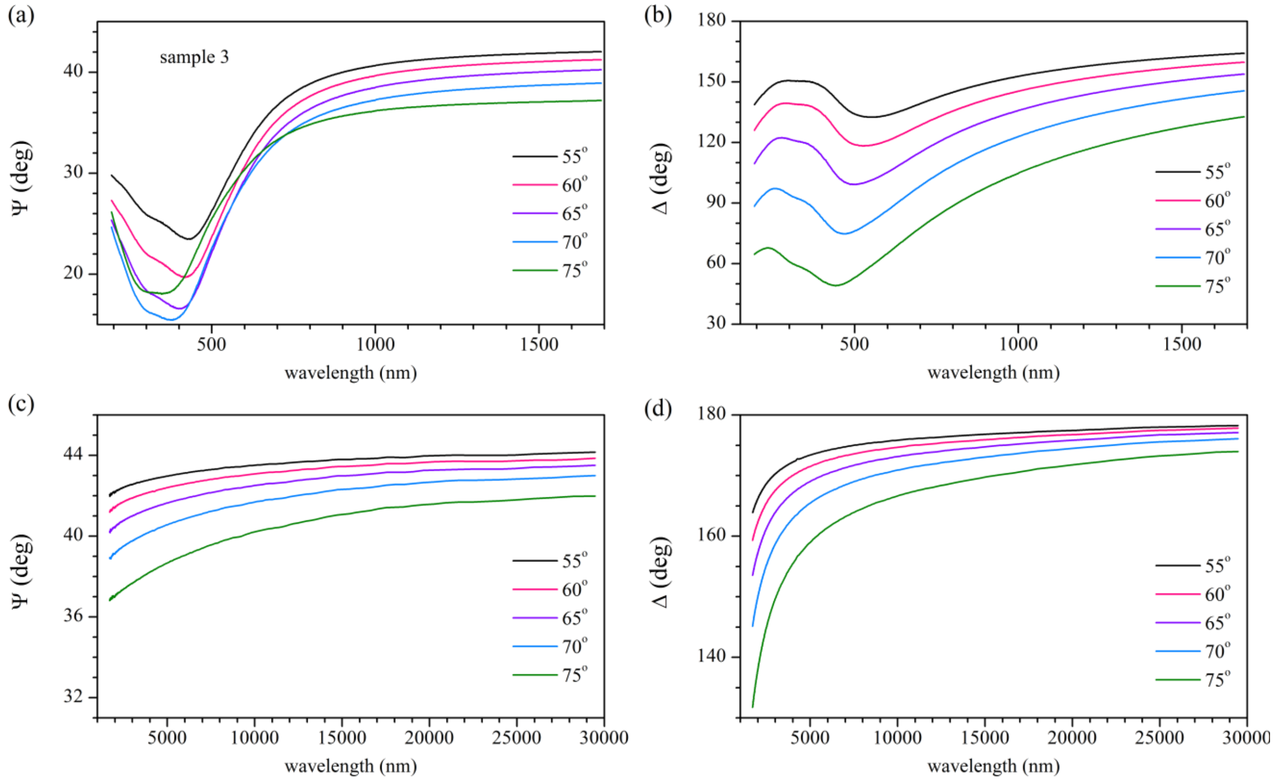

Figure S25. Ellipsometric parameters Ψ and Δ as a function of wavelength in two spectral ranges for sample number 3.

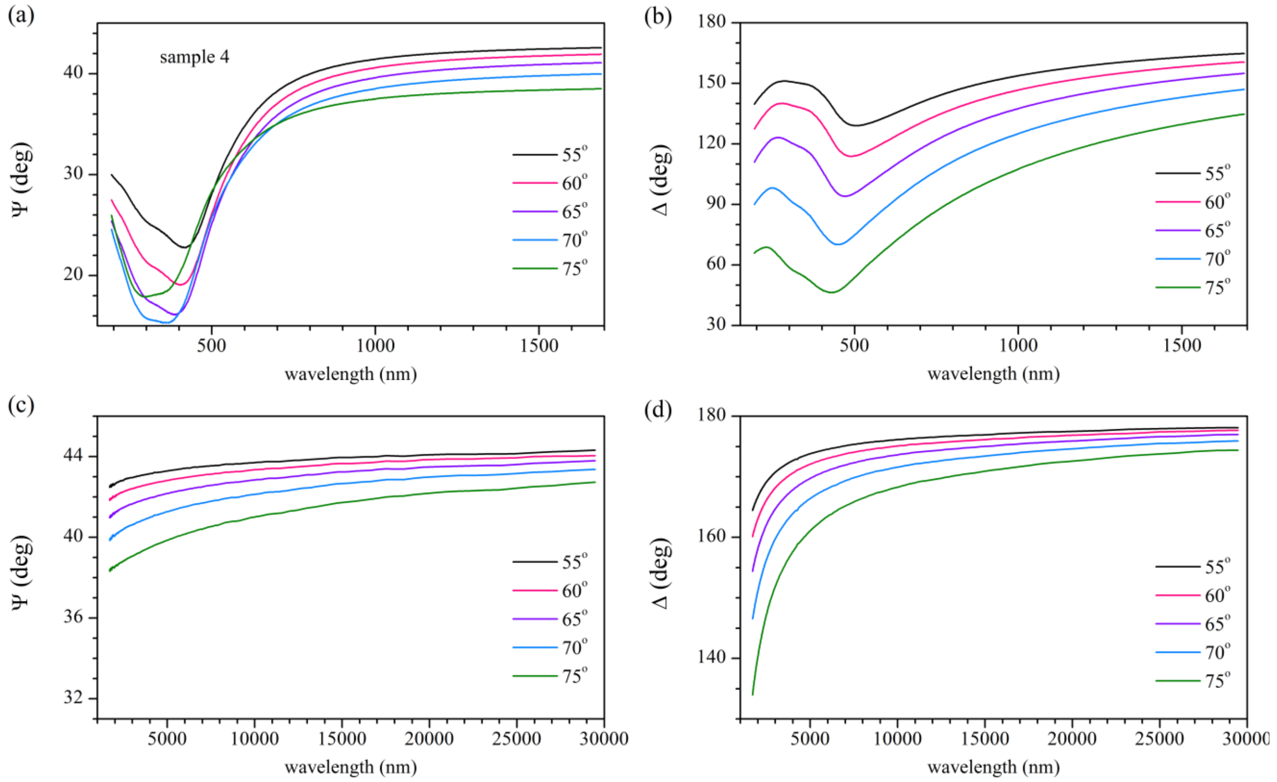

Figure S26. Ellipsometric parameters Ψ and Δ as a function of wavelength in two spectral ranges for sample number 4.

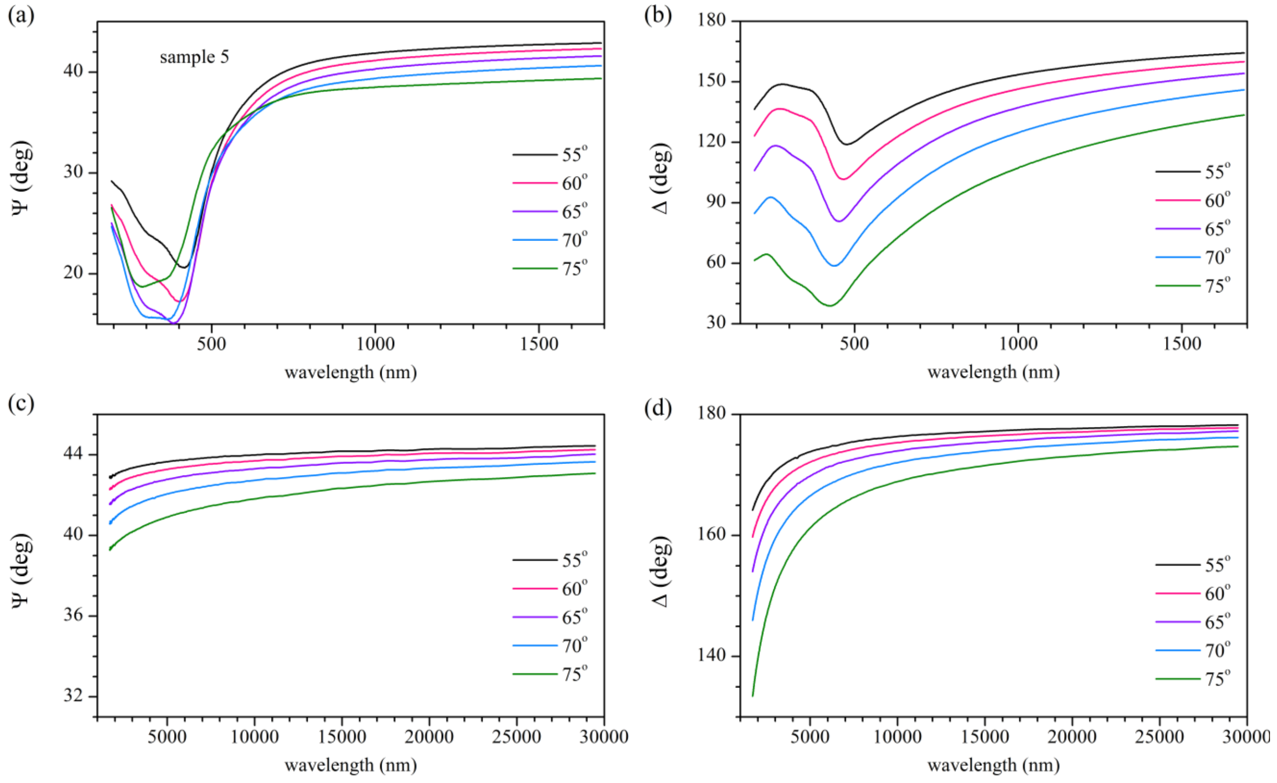

Figure S27. Ellipsometric parameters Ψ and Δ as a function of wavelength in two spectral ranges for sample number 5.

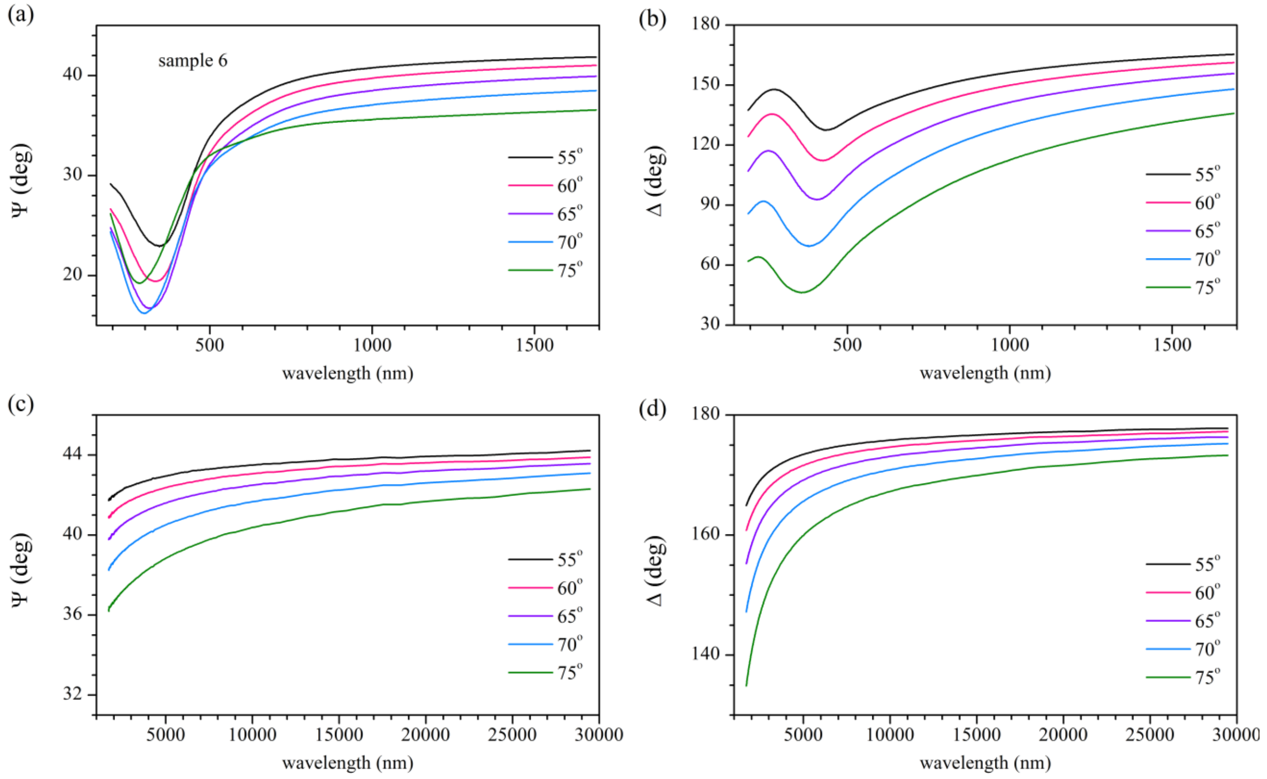

Figure S28. Ellipsometric parameters Ψ and Δ as a function of wavelength in two spectral ranges for sample number 6.

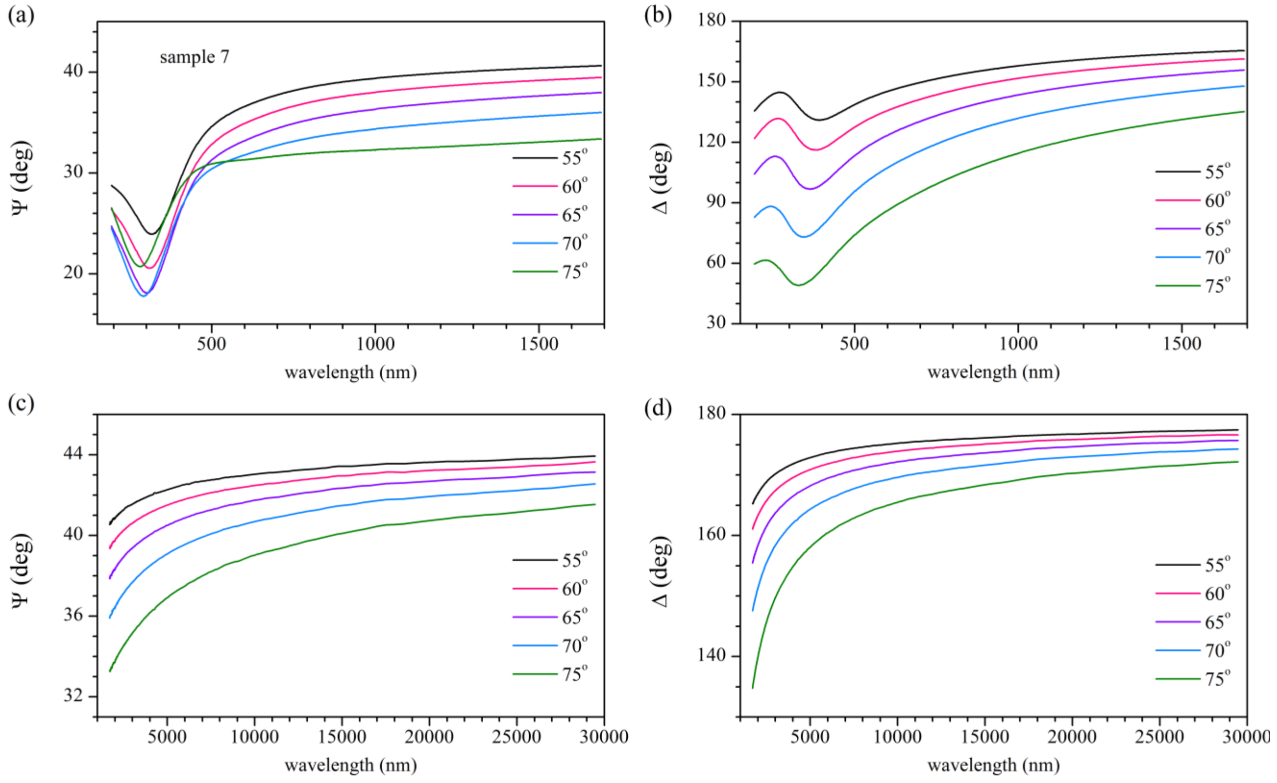

Figure S29. Ellipsometric parameters Ψ and Δ as a function of wavelength in two spectral ranges for sample number 7.

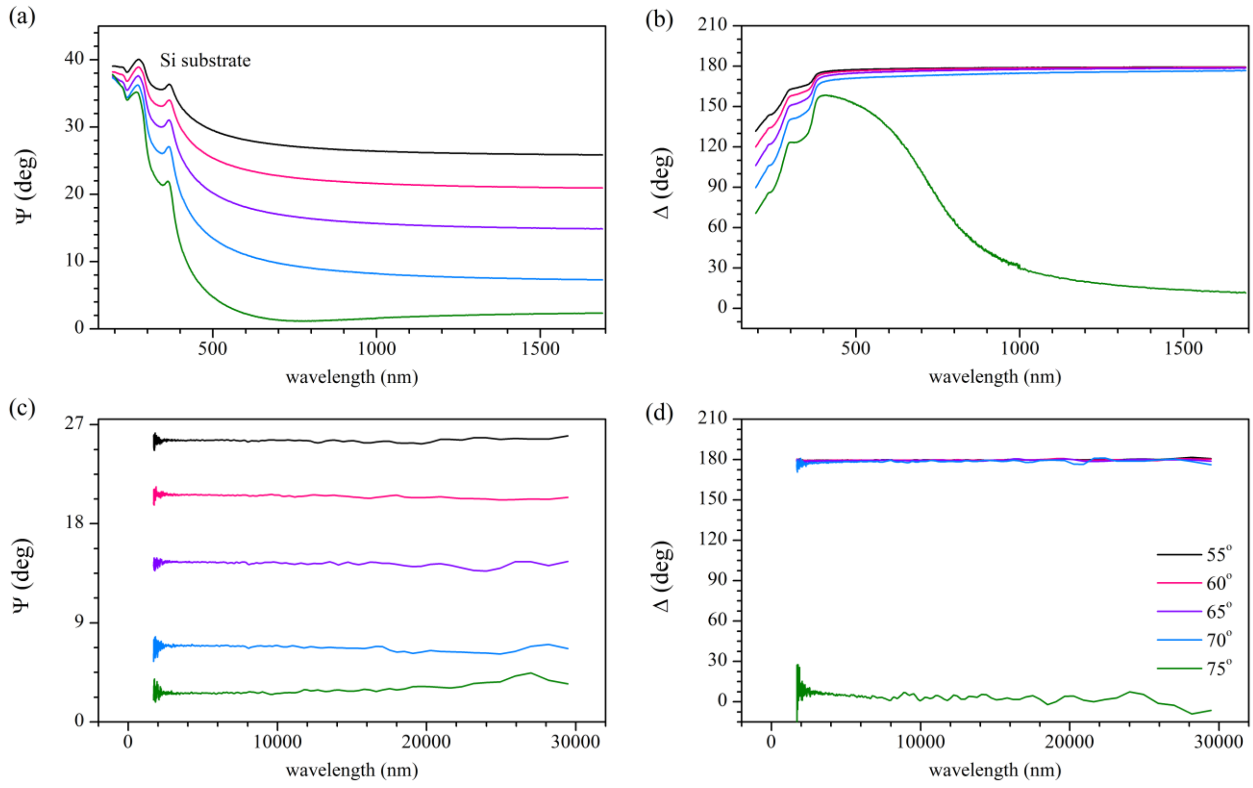

Figure S30. Ellipsometric parameters Ψ and Δ as a function of wavelength in two spectral ranges for Si substrate.

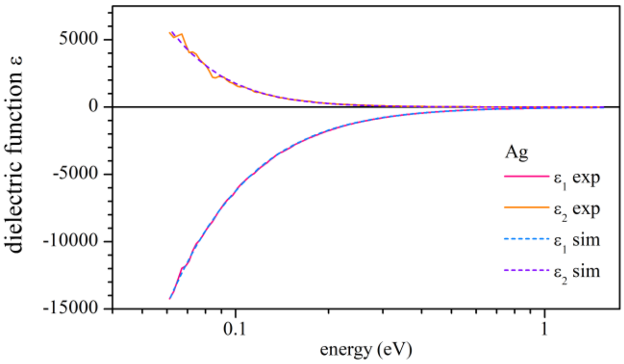

Figure S31. Extracted and simulated dielectric function in the low-energy range for freshly evaporated 300 nm thick Ag film on glass.

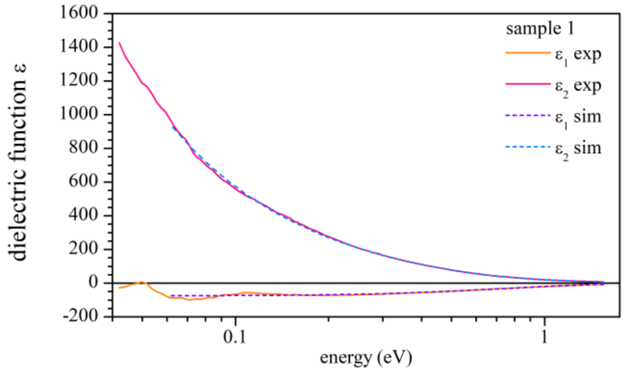

Figure S32. Extracted and simulated dielectric function in the low-energy range for sample number 1.

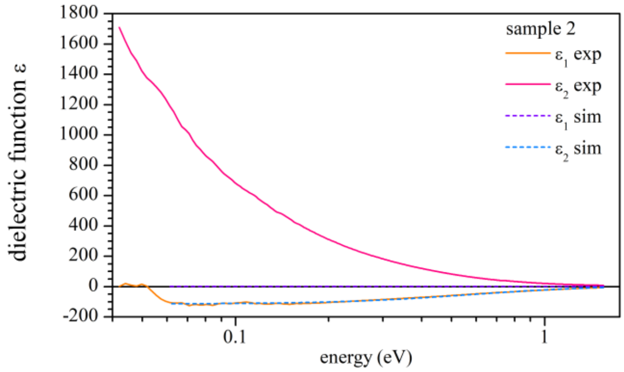

Figure S33. Extracted and simulated dielectric function in the low-energy range for sample number 2.

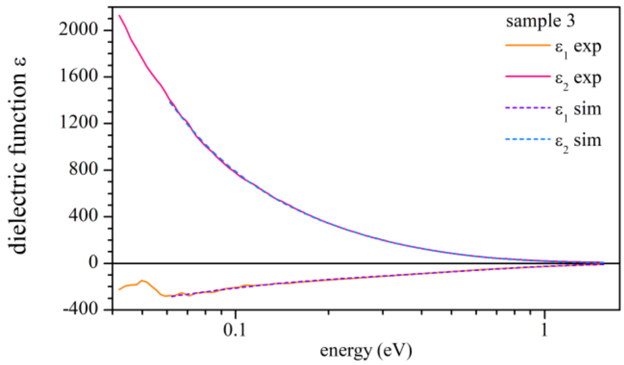

Figure S34. Extracted and simulated dielectric function in the low-energy range for sample number 3.

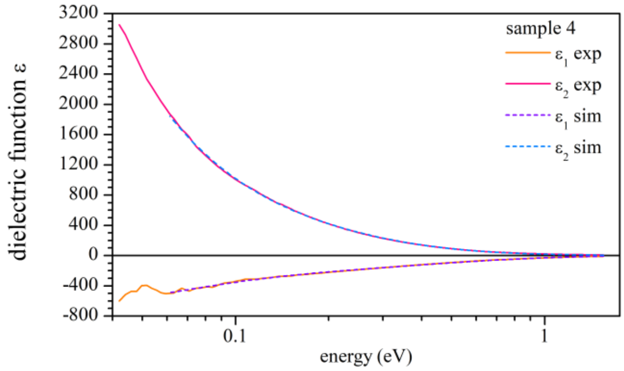

Figure S35. Extracted and simulated dielectric function in the low-energy range for sample number 4.

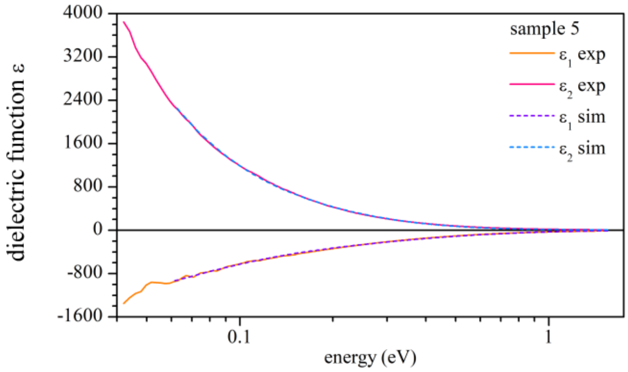

Figure S36. Extracted and simulated dielectric function in the low-energy range for sample number 5.

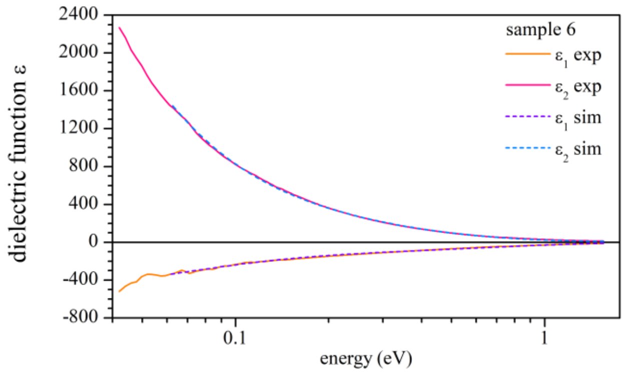

Figure S37. Extracted and simulated dielectric function in the low-energy range for sample number 6.

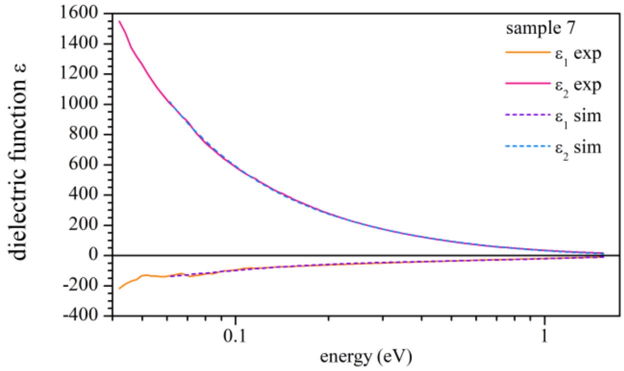

Figure S38. Extracted and simulated dielectric function in the low-energy range for sample number 7.
